# Supplementary material for: Electric field causes volumetric changes in the human brain
Source: eLife. 2019 Oct 23;8:e49115. doi: 10.7554/eLife.49115 (PMC6874416; doi:10.7554/eLife.49115)
Supplement: Supplementary file 1. — One sample t-test in each ROIs. The table indicates t, uncorrected p, Cohen’s d effect size, and FDR corrected p values. This sample is a sub-cohort of a recent publication of 331 subjects (Ousdal et al., 2019). In contrast with that publication we included patients with RUL only electrode placement. [file elife-49115-supp1.docx]

Volume changes

|  | X | roi | t | p | mean | d | BHFDR |
| --- | --- | --- | --- | --- | --- | --- | --- |
| 1 | 1.0000 | Δ VOLLeft.Cerebellum.Cortex | 0.5245 | 0.6007 | 0.0006 | 0.0427 | 0.6007 |
| 2 | 2.0000 | Δ VOLLeft.Thalamus.Proper | 10.5829 | 0.0000 | 0.0100 | 0.8612 | 0.0000 |
| 3 | 3.0000 | Δ VOLLeft.Caudate | 6.7938 | 0.0000 | 0.0118 | 0.5529 | 0.0000 |
| 4 | 4.0000 | Δ VOLLeft.Putamen | 4.9404 | 0.0000 | 0.0088 | 0.4020 | 0.0000 |
| 5 | 5.0000 | Δ VOLLeft.Pallidum | 8.7126 | 0.0000 | 0.0089 | 0.7090 | 0.0000 |
| 6 | 6.0000 | Δ VOLBrain.Stem | 1.9608 | 0.0518 | 0.0016 | 0.1596 | 0.0530 |
| 7 | 7.0000 | Δ VOLLeft.Hippocampus | 9.0893 | 0.0000 | 0.0157 | 0.7497 | 0.0000 |
| 8 | 8.0000 | Δ VOLLeft.Amygdala | 12.7007 | 0.0000 | 0.0231 | 1.0336 | 0.0000 |
| 9 | 9.0000 | Δ VOLLeft.Accumbens.area | 5.9768 | 0.0000 | 0.0112 | 0.4864 | 0.0000 |
| 10 | 10.0000 | Δ VOLLeft.VentralDC | 5.3281 | 0.0000 | 0.0043 | 0.4336 | 0.0000 |
| 11 | 11.0000 | Δ VOLRight.Cerebellum.Cortex | 0.5627 | 0.5745 | 0.0006 | 0.0458 | 0.5813 |
| 12 | 12.0000 | Δ VOLRight.Thalamus.Proper | 15.0997 | 0.0000 | 0.0137 | 1.2288 | 0.0000 |
| 13 | 13.0000 | Δ VOLRight.Caudate | 9.4496 | 0.0000 | 0.0138 | 0.7690 | 0.0000 |
| 14 | 14.0000 | Δ VOLRight.Putamen | 12.1218 | 0.0000 | 0.0136 | 0.9865 | 0.0000 |
| 15 | 15.0000 | Δ VOLRight.Pallidum | 13.5065 | 0.0000 | 0.0125 | 1.0991 | 0.0000 |
| 16 | 16.0000 | Δ VOLRight.Hippocampus | 16.3659 | 0.0000 | 0.0270 | 1.3498 | 0.0000 |
| 17 | 17.0000 | Δ VOLRight.Amygdala | 21.7232 | 0.0000 | 0.0518 | 1.7678 | 0.0000 |
| 18 | 18.0000 | Δ VOLRight.Accumbens.area | 12.0922 | 0.0000 | 0.0214 | 0.9840 | 0.0000 |
| 19 | 19.0000 | Δ VOLRight.VentralDC | 7.7591 | 0.0000 | 0.0059 | 0.6314 | 0.0000 |
| 20 | 20.0000 | Δ VOLctx.lh.bankssts | 7.6089 | 0.0000 | 0.0113 | 0.6192 | 0.0000 |
| 21 | 21.0000 | Δ VOLctx.lh.caudalanteriorcingulate | 11.2581 | 0.0000 | 0.0191 | 0.9162 | 0.0000 |
| 22 | 22.0000 | Δ VOLctx.lh.caudalmiddlefrontal | 8.2731 | 0.0000 | 0.0104 | 0.6733 | 0.0000 |
| 23 | 23.0000 | Δ VOLctx.lh.cuneus | 5.9459 | 0.0000 | 0.0055 | 0.4839 | 0.0000 |
| 24 | 24.0000 | Δ VOLctx.lh.entorhinal | 7.5440 | 0.0000 | 0.0186 | 0.6139 | 0.0000 |
| 25 | 25.0000 | Δ VOLctx.lh.fusiform | 9.5890 | 0.0000 | 0.0115 | 0.7803 | 0.0000 |
| 26 | 26.0000 | Δ VOLctx.lh.inferiorparietal | 8.6539 | 0.0000 | 0.0120 | 0.7042 | 0.0000 |
| 27 | 27.0000 | Δ VOLctx.lh.inferiortemporal | 8.2009 | 0.0000 | 0.0130 | 0.6674 | 0.0000 |
| 28 | 28.0000 | Δ VOLctx.lh.isthmuscingulate | 3.2406 | 0.0015 | 0.0037 | 0.2637 | 0.0016 |
| 29 | 29.0000 | Δ VOLctx.lh.lateraloccipital | 7.4697 | 0.0000 | 0.0086 | 0.6079 | 0.0000 |
| 30 | 30.0000 | Δ VOLctx.lh.lateralorbitofrontal | 3.6184 | 0.0004 | 0.0048 | 0.2945 | 0.0004 |
| 31 | 31.0000 | Δ VOLctx.lh.lingual | 6.4193 | 0.0000 | 0.0053 | 0.5224 | 0.0000 |
| 32 | 32.0000 | Δ VOLctx.lh.medialorbitofrontal | 3.3268 | 0.0011 | 0.0047 | 0.2707 | 0.0012 |
| 33 | 33.0000 | Δ VOLctx.lh.middletemporal | 5.3903 | 0.0000 | 0.0098 | 0.4387 | 0.0000 |
| 34 | 34.0000 | Δ VOLctx.lh.parahippocampal | 9.0527 | 0.0000 | 0.0143 | 0.7367 | 0.0000 |
| 35 | 35.0000 | Δ VOLctx.lh.paracentral | 9.9337 | 0.0000 | 0.0120 | 0.8084 | 0.0000 |
| 36 | 36.0000 | Δ VOLctx.lh.parsopercularis | 9.9665 | 0.0000 | 0.0123 | 0.8111 | 0.0000 |
| 37 | 37.0000 | Δ VOLctx.lh.parsorbitalis | 2.6751 | 0.0083 | 0.0048 | 0.2177 | 0.0087 |
| 38 | 38.0000 | Δ VOLctx.lh.parstriangularis | 6.5027 | 0.0000 | 0.0091 | 0.5292 | 0.0000 |
| 39 | 39.0000 | Δ VOLctx.lh.pericalcarine | 5.7835 | 0.0000 | 0.0053 | 0.4707 | 0.0000 |
| 40 | 40.0000 | Δ VOLctx.lh.postcentral | 6.7211 | 0.0000 | 0.0064 | 0.5470 | 0.0000 |
| 41 | 41.0000 | Δ VOLctx.lh.posteriorcingulate | 6.7536 | 0.0000 | 0.0078 | 0.5496 | 0.0000 |
| 42 | 42.0000 | Δ VOLctx.lh.precentral | 9.5193 | 0.0000 | 0.0098 | 0.7747 | 0.0000 |
| 43 | 43.0000 | Δ VOLctx.lh.precuneus | 7.0412 | 0.0000 | 0.0069 | 0.5730 | 0.0000 |
| 44 | 44.0000 | Δ VOLctx.lh.rostralanteriorcingulate | 5.9032 | 0.0000 | 0.0098 | 0.4804 | 0.0000 |
| 45 | 45.0000 | Δ VOLctx.lh.rostralmiddlefrontal | 5.0290 | 0.0000 | 0.0063 | 0.4093 | 0.0000 |
| 46 | 46.0000 | Δ VOLctx.lh.superiorfrontal | 8.5848 | 0.0000 | 0.0111 | 0.6986 | 0.0000 |
| 47 | 47.0000 | Δ VOLctx.lh.superiorparietal | 8.4914 | 0.0000 | 0.0090 | 0.6910 | 0.0000 |
| 48 | 48.0000 | Δ VOLctx.lh.superiortemporal | 5.8041 | 0.0000 | 0.0084 | 0.4723 | 0.0000 |
| 49 | 49.0000 | Δ VOLctx.lh.supramarginal | 9.4186 | 0.0000 | 0.0126 | 0.7665 | 0.0000 |
| 50 | 50.0000 | Δ VOLctx.lh.frontalpole | 2.3865 | 0.0183 | 0.0070 | 0.1942 | 0.0189 |
| 51 | 51.0000 | Δ VOLctx.lh.temporalpole | 8.2492 | 0.0000 | 0.0222 | 0.6713 | 0.0000 |
| 52 | 52.0000 | Δ VOLctx.lh.transversetemporal | 4.3494 | 0.0000 | 0.0068 | 0.3540 | 0.0000 |
| 53 | 53.0000 | Δ VOLctx.rh.bankssts | 11.6196 | 0.0000 | 0.0158 | 0.9456 | 0.0000 |
| 54 | 54.0000 | Δ VOLctx.rh.caudalanteriorcingulate | 14.1234 | 0.0000 | 0.0199 | 1.1493 | 0.0000 |
| 55 | 55.0000 | Δ VOLctx.rh.caudalmiddlefrontal | 9.3625 | 0.0000 | 0.0115 | 0.7619 | 0.0000 |
| 56 | 56.0000 | Δ VOLctx.rh.cuneus | 8.2941 | 0.0000 | 0.0073 | 0.6750 | 0.0000 |
| 57 | 57.0000 | Δ VOLctx.rh.entorhinal | 13.2836 | 0.0000 | 0.0330 | 1.0810 | 0.0000 |
| 58 | 58.0000 | Δ VOLctx.rh.fusiform | 14.6356 | 0.0000 | 0.0187 | 1.1910 | 0.0000 |
| 59 | 59.0000 | Δ VOLctx.rh.inferiorparietal | 9.7739 | 0.0000 | 0.0130 | 0.7954 | 0.0000 |
| 60 | 60.0000 | Δ VOLctx.rh.inferiortemporal | 14.2156 | 0.0000 | 0.0193 | 1.1568 | 0.0000 |
| 61 | 61.0000 | Δ VOLctx.rh.isthmuscingulate | 4.1816 | 0.0000 | 0.0040 | 0.3403 | 0.0001 |
| 62 | 62.0000 | Δ VOLctx.rh.lateraloccipital | 8.2511 | 0.0000 | 0.0103 | 0.6715 | 0.0000 |
| 63 | 63.0000 | Δ VOLctx.rh.lateralorbitofrontal | 6.4489 | 0.0000 | 0.0103 | 0.5248 | 0.0000 |
| 64 | 64.0000 | Δ VOLctx.rh.lingual | 9.1881 | 0.0000 | 0.0075 | 0.7477 | 0.0000 |
| 65 | 65.0000 | Δ VOLctx.rh.medialorbitofrontal | 7.5171 | 0.0000 | 0.0115 | 0.6117 | 0.0000 |
| 66 | 66.0000 | Δ VOLctx.rh.middletemporal | 11.6007 | 0.0000 | 0.0159 | 0.9440 | 0.0000 |
| 67 | 67.0000 | Δ VOLctx.rh.parahippocampal | 12.4883 | 0.0000 | 0.0215 | 1.0197 | 0.0000 |
| 68 | 68.0000 | Δ VOLctx.rh.paracentral | 11.6424 | 0.0000 | 0.0132 | 0.9474 | 0.0000 |
| 69 | 69.0000 | Δ VOLctx.rh.parsopercularis | 9.2831 | 0.0000 | 0.0122 | 0.7555 | 0.0000 |
| 70 | 70.0000 | Δ VOLctx.rh.parsorbitalis | 4.8321 | 0.0000 | 0.0090 | 0.3932 | 0.0000 |
| 71 | 71.0000 | Δ VOLctx.rh.parstriangularis | 9.7651 | 0.0000 | 0.0124 | 0.7947 | 0.0000 |
| 72 | 72.0000 | Δ VOLctx.rh.pericalcarine | 7.9260 | 0.0000 | 0.0068 | 0.6450 | 0.0000 |
| 73 | 73.0000 | Δ VOLctx.rh.postcentral | 7.9663 | 0.0000 | 0.0081 | 0.6483 | 0.0000 |
| 74 | 74.0000 | Δ VOLctx.rh.posteriorcingulate | 8.4401 | 0.0000 | 0.0094 | 0.6868 | 0.0000 |
| 75 | 75.0000 | Δ VOLctx.rh.precentral | 9.5727 | 0.0000 | 0.0105 | 0.7790 | 0.0000 |
| 76 | 76.0000 | Δ VOLctx.rh.precuneus | 9.0177 | 0.0000 | 0.0082 | 0.7339 | 0.0000 |
| 77 | 77.0000 | Δ VOLctx.rh.rostralanteriorcingulate | 12.6172 | 0.0000 | 0.0184 | 1.0268 | 0.0000 |
| 78 | 78.0000 | Δ VOLctx.rh.rostralmiddlefrontal | 7.1486 | 0.0000 | 0.0092 | 0.5817 | 0.0000 |
| 79 | 79.0000 | Δ VOLctx.rh.superiorfrontal | 9.3432 | 0.0000 | 0.0118 | 0.7603 | 0.0000 |
| 80 | 80.0000 | Δ VOLctx.rh.superiorparietal | 7.1766 | 0.0000 | 0.0086 | 0.5840 | 0.0000 |
| 81 | 81.0000 | Δ VOLctx.rh.superiortemporal | 15.6352 | 0.0000 | 0.0216 | 1.2724 | 0.0000 |
| 82 | 82.0000 | Δ VOLctx.rh.supramarginal | 12.4385 | 0.0000 | 0.0153 | 1.0122 | 0.0000 |
| 83 | 83.0000 | Δ VOLctx.rh.frontalpole | 3.5531 | 0.0005 | 0.0089 | 0.2891 | 0.0006 |
| 84 | 84.0000 | Δ VOLctx.rh.temporalpole | 15.0638 | 0.0000 | 0.0442 | 1.2259 | 0.0000 |
| 85 | 85.0000 | Δ VOLctx.rh.transversetemporal | 9.8355 | 0.0000 | 0.0159 | 0.8004 | 0.0000 |
