# Supplementary material for: Electric field causes volumetric changes in the human brain
Source: eLife. 2019 Oct 23;8:e49115. doi: 10.7554/eLife.49115 (PMC6874416; doi:10.7554/eLife.49115)
Supplement: Supplementary file 2. — The table contains the results of 42 pairwise t-tests between the volume changes of the right and the left side of the corresponding regions. The table indicates t, uncorrected p, mean, Cohen’s d effect size and FDR corrected p values. [file elife-49115-supp2.docx]

Pairwise difference in volume change between R and L side

|  | roi | t | p | mean | d | BHFDR |
| --- | --- | --- | --- | --- | --- | --- |
| 1 | Δ VOLR-LLeft.Cerebellum.Cortex | 0.0143 | 0.9886 | 0.0000 | 0.0012 | 0.9886 |
| 2 | Δ VOLR-LLeft.Thalamus.Proper | 5.3964 | 0.0000 | 0.0038 | 0.4392 | 0.0000 |
| 3 | Δ VOLR-LLeft.Caudate | 1.3345 | 0.1841 | 0.0020 | 0.1086 | 0.2479 |
| 4 | Δ VOLR-LLeft.Putamen | 2.9475 | 0.0037 | 0.0048 | 0.2399 | 0.0082 |
| 5 | Δ VOLR-LLeft.Pallidum | 4.1348 | 0.0001 | 0.0036 | 0.3365 | 0.0002 |
| 6 | Δ VOLR-LLeft.Hippocampus | 7.5495 | 0.0000 | 0.0112 | 0.6227 | 0.0000 |
| 7 | Δ VOLR-LLeft.Amygdala | 13.5849 | 0.0000 | 0.0287 | 1.1055 | 0.0000 |
| 8 | Δ VOLR-LLeft.Accumbens.area | 4.9434 | 0.0000 | 0.0102 | 0.4023 | 0.0000 |
| 9 | Δ VOLR-LLeft.VentralDC | 2.0243 | 0.0447 | 0.0015 | 0.1647 | 0.0722 |
| 10 | Δ VOLR-Lctx.lh.bankssts | 3.0130 | 0.0030 | 0.0045 | 0.2452 | 0.0071 |
| 11 | Δ VOLR-Lctx.lh.caudalanteriorcingulate | 0.6360 | 0.5257 | 0.0009 | 0.0518 | 0.5811 |
| 12 | Δ VOLR-Lctx.lh.caudalmiddlefrontal | 0.9711 | 0.3331 | 0.0011 | 0.0790 | 0.4239 |
| 13 | Δ VOLR-Lctx.lh.cuneus | 2.4545 | 0.0153 | 0.0018 | 0.1997 | 0.0305 |
| 14 | Δ VOLR-Lctx.lh.entorhinal | 5.8721 | 0.0000 | 0.0144 | 0.4779 | 0.0000 |
| 15 | Δ VOLR-Lctx.lh.fusiform | 6.5958 | 0.0000 | 0.0073 | 0.5368 | 0.0000 |
| 16 | Δ VOLR-Lctx.lh.inferiorparietal | 0.7300 | 0.4665 | 0.0009 | 0.0594 | 0.5296 |
| 17 | Δ VOLR-Lctx.lh.inferiortemporal | 4.0768 | 0.0001 | 0.0063 | 0.3318 | 0.0002 |
| 18 | Δ VOLR-Lctx.lh.isthmuscingulate | 0.3073 | 0.7590 | 0.0003 | 0.0250 | 0.7970 |
| 19 | Δ VOLR-Lctx.lh.lateraloccipital | 1.8303 | 0.0692 | 0.0017 | 0.1489 | 0.1076 |
| 20 | Δ VOLR-Lctx.lh.lateralorbitofrontal | 4.4129 | 0.0000 | 0.0055 | 0.3591 | 0.0001 |
| 21 | Δ VOLR-Lctx.lh.lingual | 3.4261 | 0.0008 | 0.0022 | 0.2788 | 0.0020 |
| 22 | Δ VOLR-Lctx.lh.medialorbitofrontal | 5.6916 | 0.0000 | 0.0068 | 0.4632 | 0.0000 |
| 23 | Δ VOLR-Lctx.lh.middletemporal | 3.4250 | 0.0008 | 0.0061 | 0.2787 | 0.0020 |
| 24 | Δ VOLR-Lctx.lh.parahippocampal | 5.2481 | 0.0000 | 0.0073 | 0.4285 | 0.0000 |
| 25 | Δ VOLR-Lctx.lh.paracentral | 1.3198 | 0.1889 | 0.0012 | 0.1074 | 0.2479 |
| 26 | Δ VOLR-Lctx.lh.parsopercularis | -0.0817 | 0.9350 | -0.0001 | -0.0067 | 0.9578 |
| 27 | Δ VOLR-Lctx.lh.parsorbitalis | 2.2705 | 0.0246 | 0.0042 | 0.1848 | 0.0449 |
| 28 | Δ VOLR-Lctx.lh.parstriangularis | 2.3062 | 0.0225 | 0.0032 | 0.1877 | 0.0429 |
| 29 | Δ VOLR-Lctx.lh.pericalcarine | 2.1849 | 0.0304 | 0.0015 | 0.1778 | 0.0533 |
| 30 | Δ VOLR-Lctx.lh.postcentral | 1.6675 | 0.0975 | 0.0017 | 0.1357 | 0.1365 |
| 31 | Δ VOLR-Lctx.lh.posteriorcingulate | 1.7943 | 0.0748 | 0.0015 | 0.1460 | 0.1103 |
| 32 | Δ VOLR-Lctx.lh.precentral | 0.7771 | 0.4383 | 0.0007 | 0.0632 | 0.5116 |
| 33 | Δ VOLR-Lctx.lh.precuneus | 1.7855 | 0.0762 | 0.0013 | 0.1453 | 0.1103 |
| 34 | Δ VOLR-Lctx.lh.rostralanteriorcingulate | 5.3843 | 0.0000 | 0.0086 | 0.4382 | 0.0000 |
| 35 | Δ VOLR-Lctx.lh.rostralmiddlefrontal | 2.7045 | 0.0076 | 0.0028 | 0.2201 | 0.0160 |
| 36 | Δ VOLR-Lctx.lh.superiorfrontal | 0.9084 | 0.3651 | 0.0007 | 0.0739 | 0.4511 |
| 37 | Δ VOLR-Lctx.lh.superiorparietal | -0.5389 | 0.5907 | -0.0004 | -0.0439 | 0.6362 |
| 38 | Δ VOLR-Lctx.lh.superiortemporal | 8.6326 | 0.0000 | 0.0132 | 0.7025 | 0.0000 |
| 39 | Δ VOLR-Lctx.lh.supramarginal | 2.1185 | 0.0358 | 0.0027 | 0.1724 | 0.0601 |
| 40 | Δ VOLR-Lctx.lh.frontalpole | 0.7768 | 0.4385 | 0.0019 | 0.0632 | 0.5116 |
| 41 | Δ VOLR-Lctx.lh.temporalpole | 6.6295 | 0.0000 | 0.0220 | 0.5395 | 0.0000 |
| 42 | Δ VOLR-Lctx.lh.transversetemporal | 5.8831 | 0.0000 | 0.0090 | 0.4788 | 0.0000 |
|  |  |  |  |  |  |  |
