# Supplementary material for: Electric field causes volumetric changes in the human brain
Source: eLife. 2019 Oct 23;8:e49115. doi: 10.7554/eLife.49115 (PMC6874416; doi:10.7554/eLife.49115)
Supplement: Supplementary file 3. — The table indicates the t values of the corresponding clinical covariates modeled as fixed effects: volume change, age, and number of ECT. [file elife-49115-supp3.docx]

The relationship between the clinical response (percentage change) and volume changes across individuals (Δ MADRS ~ Δ Vol + Age + ECTnum + Site)

|  | roi | tVOL | pVOL | tAge | tECTnum |
| --- | --- | --- | --- | --- | --- |
| 1 | Δ MADRS ~ Δ VOLLeft.Cerebellum.Cortex | -0.4996 | 0.6181 | 5.6129 | -2.8409 |
| 2 | Δ MADRS ~ Δ VOLLeft.Thalamus.Proper | 0.6014 | 0.5486 | 5.5399 | -3.0339 |
| 3 | Δ MADRS ~ Δ VOLLeft.Caudate | -0.9727 | 0.3324 | 5.5351 | -2.6916 |
| 4 | Δ MADRS ~ Δ VOLLeft.Putamen | 0.1944 | 0.8462 | 5.5897 | -2.9713 |
| 5 | Δ MADRS ~ Δ VOLLeft.Pallidum | -0.6016 | 0.5484 | 5.6222 | -2.8859 |
| 6 | Δ MADRS ~ Δ VOLBrain.Stem | 0.0780 | 0.9380 | 5.5551 | -2.9623 |
| 7 | Δ MADRS ~ Δ VOLLeft.Hippocampus | 0.2436 | 0.8079 | 5.3838 | -2.7114 |
| 8 | Δ MADRS ~ Δ VOLLeft.Amygdala | 0.0822 | 0.9346 | 5.5736 | -2.9514 |
| 9 | Δ MADRS ~ Δ VOLLeft.Accumbens.area | -1.1617 | 0.2473 | 5.1099 | -2.7724 |
| 10 | Δ MADRS ~ Δ VOLLeft.VentralDC | -0.2002 | 0.8416 | 5.5898 | -2.8887 |
| 11 | Δ MADRS ~ Δ VOLRight.Cerebellum.Cortex | -0.0366 | 0.9708 | 5.5858 | -2.9504 |
| 12 | Δ MADRS ~ Δ VOLRight.Thalamus.Proper | 1.4615 | 0.1461 | 5.3363 | -3.2850 |
| 13 | Δ MADRS ~ Δ VOLRight.Caudate | -0.0246 | 0.9804 | 5.5218 | -2.9425 |
| 14 | Δ MADRS ~ Δ VOLRight.Putamen | -1.0967 | 0.2746 | 5.6266 | -2.8020 |
| 15 | Δ MADRS ~ Δ VOLRight.Pallidum | 0.1767 | 0.8600 | 5.4055 | -2.9293 |
| 16 | Δ MADRS ~ Δ VOLRight.Hippocampus | -0.7164 | 0.4750 | 5.4632 | -2.4620 |
| 17 | Δ MADRS ~ Δ VOLRight.Amygdala | 1.5094 | 0.1334 | 5.1476 | -3.3036 |
| 18 | Δ MADRS ~ Δ VOLRight.Accumbens.area | 0.0774 | 0.9384 | 5.5702 | -2.8683 |
| 19 | Δ MADRS ~ Δ VOLRight.VentralDC | -0.3931 | 0.6948 | 5.6024 | -2.9437 |
| 20 | Δ MADRS ~ Δ VOLctx.lh.bankssts | -0.7238 | 0.4704 | 5.6411 | -2.7428 |
| 21 | Δ MADRS ~ Δ VOLctx.lh.caudalanteriorcingulate | 1.2169 | 0.2257 | 5.3627 | -3.1468 |
| 22 | Δ MADRS ~ Δ VOLctx.lh.caudalmiddlefrontal | 0.0928 | 0.9262 | 5.5154 | -2.9549 |
| 23 | Δ MADRS ~ Δ VOLctx.lh.cuneus | 0.6429 | 0.5213 | 5.5821 | -3.0413 |
| 24 | Δ MADRS ~ Δ VOLctx.lh.entorhinal | 0.4162 | 0.6779 | 5.6001 | -3.0022 |
| 25 | Δ MADRS ~ Δ VOLctx.lh.fusiform | 1.6961 | 0.0921 | 5.3670 | -3.2505 |
| 26 | Δ MADRS ~ Δ VOLctx.lh.inferiorparietal | 0.1364 | 0.8917 | 5.3652 | -2.9423 |
| 27 | Δ MADRS ~ Δ VOLctx.lh.inferiortemporal | 0.2425 | 0.8088 | 5.4837 | -2.9730 |
| 28 | Δ MADRS ~ Δ VOLctx.lh.isthmuscingulate | 0.2110 | 0.8332 | 5.5711 | -2.9383 |
| 29 | Δ MADRS ~ Δ VOLctx.lh.lateraloccipital | 0.7252 | 0.4695 | 5.3551 | -3.0336 |
| 30 | Δ MADRS ~ Δ VOLctx.lh.lateralorbitofrontal | -0.5301 | 0.5969 | 5.5877 | -2.8830 |
| 31 | Δ MADRS ~ Δ VOLctx.lh.lingual | 1.2153 | 0.2263 | 5.4860 | -3.1695 |
| 32 | Δ MADRS ~ Δ VOLctx.lh.medialorbitofrontal | -0.3501 | 0.7268 | 5.5881 | -2.9271 |
| 33 | Δ MADRS ~ Δ VOLctx.lh.middletemporal | -0.6038 | 0.5469 | 5.6239 | -2.8140 |
| 34 | Δ MADRS ~ Δ VOLctx.lh.parahippocampal | 1.5379 | 0.1263 | 5.3899 | -3.2626 |
| 35 | Δ MADRS ~ Δ VOLctx.lh.paracentral | 0.3763 | 0.7072 | 5.4862 | -2.9448 |
| 36 | Δ MADRS ~ Δ VOLctx.lh.parsopercularis | -0.4156 | 0.6783 | 5.6035 | -2.8779 |
| 37 | Δ MADRS ~ Δ VOLctx.lh.parsorbitalis | -1.0305 | 0.3045 | 5.5172 | -2.8969 |
| 38 | Δ MADRS ~ Δ VOLctx.lh.parstriangularis | -1.2208 | 0.2242 | 5.5629 | -2.6377 |
| 39 | Δ MADRS ~ Δ VOLctx.lh.pericalcarine | 0.7751 | 0.4396 | 5.5888 | -3.0587 |
| 40 | Δ MADRS ~ Δ VOLctx.lh.postcentral | -1.0000 | 0.3190 | 5.4727 | -2.6996 |
| 41 | Δ MADRS ~ Δ VOLctx.lh.posteriorcingulate | 0.5498 | 0.5833 | 5.4460 | -3.0158 |
| 42 | Δ MADRS ~ Δ VOLctx.lh.precentral | -0.0495 | 0.9606 | 5.5712 | -2.9143 |
| 43 | Δ MADRS ~ Δ VOLctx.lh.precuneus | 0.3625 | 0.7175 | 5.4787 | -2.9521 |
| 44 | Δ MADRS ~ Δ VOLctx.lh.rostralanteriorcingulate | -0.1290 | 0.8975 | 5.5730 | -2.9618 |
| 45 | Δ MADRS ~ Δ VOLctx.lh.rostralmiddlefrontal | -1.9889 | 0.0487 | 5.1060 | -2.7949 |
| 46 | Δ MADRS ~ Δ VOLctx.lh.superiorfrontal | -0.3027 | 0.7626 | 5.5862 | -2.9033 |
| 47 | Δ MADRS ~ Δ VOLctx.lh.superiorparietal | -0.0438 | 0.9651 | 5.4356 | -2.8571 |
| 48 | Δ MADRS ~ Δ VOLctx.lh.superiortemporal | -0.3779 | 0.7061 | 5.5151 | -2.8940 |
| 49 | Δ MADRS ~ Δ VOLctx.lh.supramarginal | 0.1638 | 0.8702 | 5.5116 | -2.9469 |
| 50 | Δ MADRS ~ Δ VOLctx.lh.frontalpole | -1.0660 | 0.2882 | 5.6368 | -2.9350 |
| 51 | Δ MADRS ~ Δ VOLctx.lh.temporalpole | -0.0468 | 0.9627 | 5.5854 | -2.9437 |
| 52 | Δ MADRS ~ Δ VOLctx.lh.transversetemporal | 0.2540 | 0.7999 | 5.5916 | -2.9687 |
| 53 | Δ MADRS ~ Δ VOLctx.rh.bankssts | 0.9053 | 0.3668 | 5.0801 | -3.1088 |
| 54 | Δ MADRS ~ Δ VOLctx.rh.caudalanteriorcingulate | 0.3475 | 0.7288 | 5.2346 | -2.9811 |
| 55 | Δ MADRS ~ Δ VOLctx.rh.caudalmiddlefrontal | -1.0823 | 0.2809 | 5.6919 | -2.7464 |
| 56 | Δ MADRS ~ Δ VOLctx.rh.cuneus | 1.8884 | 0.0610 | 5.3569 | -3.3128 |
| 57 | Δ MADRS ~ Δ VOLctx.rh.entorhinal | 0.0560 | 0.9554 | 5.5400 | -2.9105 |
| 58 | Δ MADRS ~ Δ VOLctx.rh.fusiform | 1.8334 | 0.0688 | 4.8882 | -3.4592 |
| 59 | Δ MADRS ~ Δ VOLctx.rh.inferiorparietal | 0.5439 | 0.5874 | 5.0947 | -2.9941 |
| 60 | Δ MADRS ~ Δ VOLctx.rh.inferiortemporal | 1.3208 | 0.1887 | 5.0209 | -3.2240 |
| 61 | Δ MADRS ~ Δ VOLctx.rh.isthmuscingulate | -0.3354 | 0.7378 | 5.5933 | -2.9160 |
| 62 | Δ MADRS ~ Δ VOLctx.rh.lateraloccipital | 0.8522 | 0.3956 | 5.3702 | -3.0514 |
| 63 | Δ MADRS ~ Δ VOLctx.rh.lateralorbitofrontal | -0.3644 | 0.7161 | 5.5981 | -2.8076 |
| 64 | Δ MADRS ~ Δ VOLctx.rh.lingual | 1.4913 | 0.1381 | 5.4989 | -3.2735 |
| 65 | Δ MADRS ~ Δ VOLctx.rh.medialorbitofrontal | -0.7994 | 0.4254 | 5.6489 | -2.7635 |
| 66 | Δ MADRS ~ Δ VOLctx.rh.middletemporal | -0.2340 | 0.8153 | 5.3303 | -2.8169 |
| 67 | Δ MADRS ~ Δ VOLctx.rh.parahippocampal | 0.9612 | 0.3381 | 5.3354 | -3.1011 |
| 68 | Δ MADRS ~ Δ VOLctx.rh.paracentral | -0.1723 | 0.8635 | 5.4405 | -2.8938 |
| 69 | Δ MADRS ~ Δ VOLctx.rh.parsopercularis | -1.2194 | 0.2247 | 5.7367 | -2.6122 |
| 70 | Δ MADRS ~ Δ VOLctx.rh.parsorbitalis | -0.4771 | 0.6340 | 5.5286 | -2.8337 |
| 71 | Δ MADRS ~ Δ VOLctx.rh.parstriangularis | -0.9698 | 0.3338 | 5.6854 | -2.7035 |
| 72 | Δ MADRS ~ Δ VOLctx.rh.pericalcarine | 0.6953 | 0.4880 | 5.5519 | -3.0529 |
| 73 | Δ MADRS ~ Δ VOLctx.rh.postcentral | -0.8104 | 0.4191 | 5.6535 | -2.7233 |
| 74 | Δ MADRS ~ Δ VOLctx.rh.posteriorcingulate | 0.2733 | 0.7850 | 5.3170 | -2.9835 |
| 75 | Δ MADRS ~ Δ VOLctx.rh.precentral | -0.5814 | 0.5619 | 5.6124 | -2.7227 |
| 76 | Δ MADRS ~ Δ VOLctx.rh.precuneus | 0.9170 | 0.3607 | 5.2108 | -3.0999 |
| 77 | Δ MADRS ~ Δ VOLctx.rh.rostralanteriorcingulate | -0.2783 | 0.7812 | 5.5346 | -2.8761 |
| 78 | Δ MADRS ~ Δ VOLctx.rh.rostralmiddlefrontal | -1.9601 | 0.0520 | 5.7845 | -2.6038 |
| 79 | Δ MADRS ~ Δ VOLctx.rh.superiorfrontal | -0.5633 | 0.5741 | 5.5987 | -2.7686 |
| 80 | Δ MADRS ~ Δ VOLctx.rh.superiorparietal | -0.0981 | 0.9220 | 5.5139 | -2.9201 |
| 81 | Δ MADRS ~ Δ VOLctx.rh.superiortemporal | 0.2295 | 0.8188 | 5.2989 | -2.8918 |
| 82 | Δ MADRS ~ Δ VOLctx.rh.supramarginal | 0.5443 | 0.5871 | 5.0334 | -3.0211 |
| 83 | Δ MADRS ~ Δ VOLctx.rh.frontalpole | -0.6669 | 0.5059 | 5.6124 | -2.8353 |
| 84 | Δ MADRS ~ Δ VOLctx.rh.temporalpole | -0.1912 | 0.8486 | 5.5429 | -2.7693 |
| 85 | Δ MADRS ~ Δ VOLctx.rh.transversetemporal | 1.1493 | 0.2524 | 5.0365 | -3.1736 |

The relationship between the clinical response (absolute change) and volume changes across individuals (Δ MADRS ~ Δ Vol +Baseline MADRS + Age + ECTnum + Site)

|  | roi | tVOL | pVOL | t_BasMADRS_ | tAge | tECTnum |
| --- | --- | --- | --- | --- | --- | --- |
| 1 | Δ MADRS ~ Δ VOLLeft.Cerebellum.Cortex | -0.952 | 0.343 | 9.544 | 5.255 | -2.942 |
| 2 | Δ MADRS ~ Δ VOLLeft.Thalamus.Proper | 0.010 | 0.992 | 9.338 | 5.606 | -3.028 |
| 3 | Δ MADRS ~ Δ VOLLeft.Caudate | -1.238 | 0.218 | 9.599 | 5.166 | -2.810 |
| 4 | Δ MADRS ~ Δ VOLLeft.Putamen | 0.081 | 0.935 | 9.469 | 5.664 | -3.093 |
| 5 | Δ MADRS ~ Δ VOLLeft.Pallidum | -1.228 | 0.222 | 9.616 | 5.632 | -2.979 |
| 6 | Δ MADRS ~ Δ VOLBrain.Stem | -0.455 | 0.650 | 9.512 | 5.674 | -3.046 |
| 7 | Δ MADRS ~ Δ VOLLeft.Hippocampus | 0.370 | 0.712 | 9.359 | 5.681 | -2.861 |
| 8 | Δ MADRS ~ Δ VOLLeft.Amygdala | 0.047 | 0.963 | 9.485 | 5.634 | -3.081 |
| 9 | Δ MADRS ~ Δ VOLLeft.Accumbens.area | -0.801 | 0.424 | 9.513 | 5.069 | -2.972 |
| 10 | Δ MADRS ~ Δ VOLLeft.VentralDC | -0.767 | 0.444 | 9.531 | 5.672 | -2.927 |
| 11 | Δ MADRS ~ Δ VOLRight.Cerebellum.Cortex | -0.335 | 0.738 | 9.500 | 5.649 | -3.056 |
| 12 | Δ MADRS ~ Δ VOLRight.Thalamus.Proper | 0.741 | 0.460 | 9.351 | 5.326 | -3.203 |
| 13 | Δ MADRS ~ Δ VOLRight.Caudate | -0.417 | 0.677 | 9.499 | 5.591 | -3.035 |
| 14 | Δ MADRS ~ Δ VOLRight.Putamen | -1.676 | 0.096 | 9.685 | 4.948 | -2.944 |
| 15 | Δ MADRS ~ Δ VOLRight.Pallidum | -0.322 | 0.748 | 9.497 | 5.501 | -2.952 |
| 16 | Δ MADRS ~ Δ VOLRight.Hippocampus | -0.643 | 0.522 | 9.255 | 5.563 | -2.608 |
| 17 | Δ MADRS ~ Δ VOLRight.Amygdala | 1.137 | 0.257 | 9.508 | 5.528 | -3.308 |
| 18 | Δ MADRS ~ Δ VOLRight.Accumbens.area | -0.105 | 0.917 | 9.486 | 5.557 | -2.956 |
| 19 | Δ MADRS ~ Δ VOLRight.VentralDC | -1.085 | 0.280 | 9.558 | 5.711 | -3.050 |
| 20 | Δ MADRS ~ Δ VOLctx.lh.bankssts | -1.310 | 0.192 | 9.615 | 4.797 | -2.840 |
| 21 | Δ MADRS ~ Δ VOLctx.lh.caudalanteriorcingulate | 1.042 | 0.299 | 9.378 | 5.863 | -3.225 |
| 22 | Δ MADRS ~ Δ VOLctx.lh.caudalmiddlefrontal | -0.390 | 0.697 | 9.488 | 5.444 | -3.030 |
| 23 | Δ MADRS ~ Δ VOLctx.lh.cuneus | 0.308 | 0.759 | 9.425 | 5.761 | -3.110 |
| 24 | Δ MADRS ~ Δ VOLctx.lh.entorhinal | 0.407 | 0.685 | 9.492 | 5.807 | -3.128 |
| 25 | Δ MADRS ~ Δ VOLctx.lh.fusiform | 1.064 | 0.289 | 9.165 | 5.976 | -3.226 |
| 26 | Δ MADRS ~ Δ VOLctx.lh.inferiorparietal | -0.191 | 0.848 | 9.472 | 5.379 | -3.028 |
| 27 | Δ MADRS ~ Δ VOLctx.lh.inferiortemporal | -0.124 | 0.901 | 9.403 | 5.540 | -3.056 |
| 28 | Δ MADRS ~ Δ VOLctx.lh.isthmuscingulate | -0.169 | 0.866 | 9.483 | 5.581 | -2.986 |
| 29 | Δ MADRS ~ Δ VOLctx.lh.lateraloccipital | 0.573 | 0.568 | 9.372 | 5.695 | -3.138 |
| 30 | Δ MADRS ~ Δ VOLctx.lh.lateralorbitofrontal | -0.725 | 0.470 | 9.528 | 5.510 | -3.006 |
| 31 | Δ MADRS ~ Δ VOLctx.lh.lingual | 0.160 | 0.873 | 9.274 | 5.709 | -3.071 |
| 32 | Δ MADRS ~ Δ VOLctx.lh.medialorbitofrontal | -0.199 | 0.842 | 9.498 | 5.684 | -3.072 |
| 33 | Δ MADRS ~ Δ VOLctx.lh.middletemporal | -0.901 | 0.369 | 9.511 | 5.089 | -2.940 |
| 34 | Δ MADRS ~ Δ VOLctx.lh.parahippocampal | 1.334 | 0.184 | 9.494 | 5.726 | -3.332 |
| 35 | Δ MADRS ~ Δ VOLctx.lh.paracentral | -0.202 | 0.840 | 9.322 | 5.518 | -2.894 |
| 36 | Δ MADRS ~ Δ VOLctx.lh.parsopercularis | -0.853 | 0.395 | 9.537 | 5.148 | -2.986 |
| 37 | Δ MADRS ~ Δ VOLctx.lh.parsorbitalis | -1.211 | 0.228 | 9.590 | 5.013 | -3.051 |
| 38 | Δ MADRS ~ Δ VOLctx.lh.parstriangularis | -1.404 | 0.163 | 9.588 | 4.779 | -2.778 |
| 39 | Δ MADRS ~ Δ VOLctx.lh.pericalcarine | -0.081 | 0.936 | 9.423 | 5.678 | -3.061 |
| 40 | Δ MADRS ~ Δ VOLctx.lh.postcentral | -1.121 | 0.264 | 9.522 | 4.982 | -2.850 |
| 41 | Δ MADRS ~ Δ VOLctx.lh.posteriorcingulate | 0.266 | 0.791 | 9.381 | 5.698 | -3.065 |
| 42 | Δ MADRS ~ Δ VOLctx.lh.precentral | -0.615 | 0.540 | 9.453 | 5.304 | -2.969 |
| 43 | Δ MADRS ~ Δ VOLctx.lh.precuneus | -0.292 | 0.770 | 9.351 | 5.417 | -2.903 |
| 44 | Δ MADRS ~ Δ VOLctx.lh.rostralanteriorcingulate | -0.091 | 0.928 | 9.489 | 5.649 | -3.098 |
| 45 | Δ MADRS ~ Δ VOLctx.lh.rostralmiddlefrontal | -2.041 | 0.043 | 9.585 | 4.662 | -2.973 |
| 46 | Δ MADRS ~ Δ VOLctx.lh.superiorfrontal | -0.839 | 0.403 | 9.517 | 5.364 | -2.980 |
| 47 | Δ MADRS ~ Δ VOLctx.lh.superiorparietal | -0.615 | 0.540 | 9.478 | 5.366 | -2.862 |
| 48 | Δ MADRS ~ Δ VOLctx.lh.superiortemporal | -0.781 | 0.436 | 9.496 | 4.891 | -3.021 |
| 49 | Δ MADRS ~ Δ VOLctx.lh.supramarginal | -0.290 | 0.772 | 9.465 | 5.397 | -3.016 |
| 50 | Δ MADRS ~ Δ VOLctx.lh.frontalpole | -1.292 | 0.198 | 9.471 | 5.206 | -3.092 |
| 51 | Δ MADRS ~ Δ VOLctx.lh.temporalpole | -0.262 | 0.794 | 9.481 | 5.609 | -3.058 |
| 52 | Δ MADRS ~ Δ VOLctx.lh.transversetemporal | -0.297 | 0.767 | 9.481 | 5.462 | -3.018 |
| 53 | Δ MADRS ~ Δ VOLctx.rh.bankssts | 0.509 | 0.611 | 9.404 | 5.360 | -3.139 |
| 54 | Δ MADRS ~ Δ VOLctx.rh.caudalanteriorcingulate | 0.113 | 0.910 | 9.494 | 5.441 | -3.068 |
| 55 | Δ MADRS ~ Δ VOLctx.rh.caudalmiddlefrontal | -1.898 | 0.060 | 9.656 | 5.561 | -2.763 |
| 56 | Δ MADRS ~ Δ VOLctx.rh.cuneus | 1.097 | 0.275 | 9.265 | 6.054 | -3.237 |
| 57 | Δ MADRS ~ Δ VOLctx.rh.entorhinal | -0.139 | 0.890 | 9.472 | 5.602 | -3.012 |
| 58 | Δ MADRS ~ Δ VOLctx.rh.fusiform | 1.236 | 0.219 | 9.095 | 5.235 | -3.351 |
| 59 | Δ MADRS ~ Δ VOLctx.rh.inferiorparietal | -0.121 | 0.904 | 9.414 | 5.358 | -3.103 |
| 60 | Δ MADRS ~ Δ VOLctx.rh.inferiortemporal | 0.691 | 0.491 | 9.135 | 5.238 | -3.185 |
| 61 | Δ MADRS ~ Δ VOLctx.rh.isthmuscingulate | -0.729 | 0.467 | 9.521 | 5.395 | -3.038 |
| 62 | Δ MADRS ~ Δ VOLctx.rh.lateraloccipital | -0.028 | 0.978 | 9.353 | 5.569 | -3.088 |
| 63 | Δ MADRS ~ Δ VOLctx.rh.lateralorbitofrontal | -0.628 | 0.531 | 9.519 | 5.460 | -2.907 |
| 64 | Δ MADRS ~ Δ VOLctx.rh.lingual | 0.685 | 0.494 | 9.280 | 5.957 | -3.164 |
| 65 | Δ MADRS ~ Δ VOLctx.rh.medialorbitofrontal | -0.723 | 0.471 | 9.462 | 5.556 | -2.929 |
| 66 | Δ MADRS ~ Δ VOLctx.rh.middletemporal | -0.865 | 0.389 | 9.544 | 5.369 | -2.796 |
| 67 | Δ MADRS ~ Δ VOLctx.rh.parahippocampal | 0.424 | 0.673 | 9.347 | 5.717 | -3.072 |
| 68 | Δ MADRS ~ Δ VOLctx.rh.paracentral | -0.891 | 0.375 | 9.551 | 5.619 | -2.914 |
| 69 | Δ MADRS ~ Δ VOLctx.rh.parsopercularis | -1.735 | 0.085 | 9.704 | 5.524 | -2.655 |
| 70 | Δ MADRS ~ Δ VOLctx.rh.parsorbitalis | -1.078 | 0.283 | 9.574 | 5.155 | -2.907 |
| 71 | Δ MADRS ~ Δ VOLctx.rh.parstriangularis | -1.611 | 0.109 | 9.667 | 5.407 | -2.749 |
| 72 | Δ MADRS ~ Δ VOLctx.rh.pericalcarine | -0.171 | 0.865 | 9.442 | 5.625 | -3.031 |
| 73 | Δ MADRS ~ Δ VOLctx.rh.postcentral | -1.273 | 0.205 | 9.595 | 5.816 | -2.748 |
| 74 | Δ MADRS ~ Δ VOLctx.rh.posteriorcingulate | 0.035 | 0.972 | 9.489 | 5.530 | -3.087 |
| 75 | Δ MADRS ~ Δ VOLctx.rh.precentral | -1.513 | 0.132 | 9.674 | 5.517 | -2.625 |
| 76 | Δ MADRS ~ Δ VOLctx.rh.precuneus | -0.050 | 0.960 | 9.391 | 5.460 | -3.047 |
| 77 | Δ MADRS ~ Δ VOLctx.rh.rostralanteriorcingulate | -0.679 | 0.498 | 9.489 | 5.434 | -2.961 |
| 78 | Δ MADRS ~ Δ VOLctx.rh.rostralmiddlefrontal | -2.549 | 0.012 | 9.768 | 5.470 | -2.679 |
| 79 | Δ MADRS ~ Δ VOLctx.rh.superiorfrontal | -1.204 | 0.231 | 9.549 | 5.454 | -2.773 |
| 80 | Δ MADRS ~ Δ VOLctx.rh.superiorparietal | -0.800 | 0.425 | 9.516 | 5.479 | -2.960 |
| 81 | Δ MADRS ~ Δ VOLctx.rh.superiortemporal | -0.550 | 0.583 | 9.476 | 5.303 | -2.786 |
| 82 | Δ MADRS ~ Δ VOLctx.rh.supramarginal | -0.459 | 0.647 | 9.457 | 5.327 | -2.930 |
| 83 | Δ MADRS ~ Δ VOLctx.rh.frontalpole | -1.344 | 0.181 | 9.583 | 5.183 | -2.914 |
| 84 | Δ MADRS ~ Δ VOLctx.rh.temporalpole | -0.630 | 0.530 | 9.490 | 5.335 | -2.798 |
| 85 | Δ MADRS ~ Δ VOLctx.rh.transversetemporal | 0.240 | 0.810 | 9.370 | 5.487 | -3.070 |
