# Supplementary material for: Electric field causes volumetric changes in the human brain
Source: eLife. 2019 Oct 23;8:e49115. doi: 10.7554/eLife.49115 (PMC6874416; doi:10.7554/eLife.49115)
Supplement: Supplementary file 4. — The table indicates the t values of the corresponding clinical covariates modeled as fixed effects: electric field, age, and number of ECT. [file elife-49115-supp4.docx]

The relationship between clinical response (percentage change) and EF across individuals (Δ MADRS ~ EF + Age + ECTnum + Site)

|  | roi | tEF | pEF | tAge | tECTnum |
| --- | --- | --- | --- | --- | --- |
| 1 | Δ MADRS ~ EFLeft.Cerebellum.Cortex | 1.3516 | 0.1787 | 5.3362 | -2.9932 |
| 2 | Δ MADRS ~ EFLeft.Thalamus.Proper | 0.8783 | 0.3813 | 5.6445 | -3.0320 |
| 3 | Δ MADRS ~ EFLeft.Caudate | 0.4661 | 0.6419 | 5.5098 | -2.9904 |
| 4 | Δ MADRS ~ EFLeft.Putamen | 0.8759 | 0.3826 | 5.6312 | -3.0171 |
| 5 | Δ MADRS ~ EFLeft.Pallidum | 1.1551 | 0.2500 | 5.7127 | -3.0332 |
| 6 | Δ MADRS ~ EFBrain.Stem | 0.8996 | 0.3699 | 5.5420 | -2.9615 |
| 7 | Δ MADRS ~ EFLeft.Hippocampus | 1.2165 | 0.2258 | 5.6648 | -2.9576 |
| 8 | Δ MADRS ~ EFLeft.Amygdala | 1.0714 | 0.2858 | 5.7090 | -2.9743 |
| 9 | Δ MADRS ~ EFLeft.Accumbens.area | 0.8968 | 0.3713 | 5.5454 | -3.0450 |
| 10 | Δ MADRS ~ EFLeft.VentralDC | 0.8683 | 0.3867 | 5.6287 | -2.9751 |
| 11 | Δ MADRS ~ EFRight.Cerebellum.Cortex | 0.9875 | 0.3251 | 5.4111 | -2.9886 |
| 12 | Δ MADRS ~ EFRight.Thalamus.Proper | 0.5893 | 0.5566 | 5.4818 | -3.0056 |
| 13 | Δ MADRS ~ EFRight.Caudate | 0.6738 | 0.5016 | 5.3063 | -2.9863 |
| 14 | Δ MADRS ~ EFRight.Putamen | 0.6475 | 0.5184 | 5.4451 | -2.9704 |
| 15 | Δ MADRS ~ EFRight.Pallidum | 0.5802 | 0.5627 | 5.4664 | -2.9926 |
| 16 | Δ MADRS ~ EFRight.Hippocampus | 0.6835 | 0.4954 | 5.2478 | -2.9474 |
| 17 | Δ MADRS ~ EFRight.Amygdala | 0.3874 | 0.6990 | 5.4204 | -2.9439 |
| 18 | Δ MADRS ~ EFRight.Accumbens.area | 0.3876 | 0.6989 | 5.0600 | -2.9816 |
| 19 | Δ MADRS ~ EFRight.VentralDC | 0.6604 | 0.5101 | 5.4074 | -2.9732 |
| 20 | Δ MADRS ~ EFctx.lh.bankssts | 0.8611 | 0.3907 | 5.6521 | -2.9588 |
| 21 | Δ MADRS ~ EFctx.lh.caudalanteriorcingulate | 0.8251 | 0.4107 | 5.5545 | -3.0185 |
| 22 | Δ MADRS ~ EFctx.lh.caudalmiddlefrontal | 1.7234 | 0.0870 | 5.5849 | -3.0935 |
| 23 | Δ MADRS ~ EFctx.lh.cuneus | 1.1248 | 0.2626 | 5.5896 | -2.9989 |
| 24 | Δ MADRS ~ EFctx.lh.entorhinal | 0.9818 | 0.3279 | 5.1629 | -3.0469 |
| 25 | Δ MADRS ~ EFctx.lh.fusiform | 0.7246 | 0.4699 | 5.3341 | -2.9969 |
| 26 | Δ MADRS ~ EFctx.lh.inferiorparietal | 1.2372 | 0.2181 | 5.6178 | -3.0306 |
| 27 | Δ MADRS ~ EFctx.lh.inferiortemporal | 0.7907 | 0.4304 | 5.6349 | -2.9838 |
| 28 | Δ MADRS ~ EFctx.lh.isthmuscingulate | 1.2497 | 0.2135 | 5.5542 | -3.0068 |
| 29 | Δ MADRS ~ EFctx.lh.lateraloccipital | 0.6946 | 0.4885 | 5.5630 | -2.9939 |
| 30 | Δ MADRS ~ EFctx.lh.lateralorbitofrontal | 1.2830 | 0.2016 | 5.6728 | -2.9776 |
| 31 | Δ MADRS ~ EFctx.lh.lingual | -0.0992 | 0.9211 | 5.4741 | -2.9728 |
| 32 | Δ MADRS ~ EFctx.lh.medialorbitofrontal | 1.0597 | 0.2911 | 5.4500 | -2.9399 |
| 33 | Δ MADRS ~ EFctx.lh.middletemporal | 1.3588 | 0.1764 | 5.7274 | -3.0252 |
| 34 | Δ MADRS ~ EFctx.lh.parahippocampal | 1.0622 | 0.2900 | 5.2664 | -2.9874 |
| 35 | Δ MADRS ~ EFctx.lh.paracentral | 1.5686 | 0.1190 | 5.3424 | -3.0830 |
| 36 | Δ MADRS ~ EFctx.lh.parsopercularis | 0.8736 | 0.3838 | 5.6654 | -2.9925 |
| 37 | Δ MADRS ~ EFctx.lh.parsorbitalis | 0.1145 | 0.9090 | 5.2936 | -2.9698 |
| 38 | Δ MADRS ~ EFctx.lh.parstriangularis | 0.6089 | 0.5436 | 5.6063 | -2.9876 |
| 39 | Δ MADRS ~ EFctx.lh.pericalcarine | -0.1227 | 0.9025 | 5.5874 | -2.9741 |
| 40 | Δ MADRS ~ EFctx.lh.postcentral | 1.5423 | 0.1252 | 5.4710 | -3.0388 |
| 41 | Δ MADRS ~ EFctx.lh.posteriorcingulate | 1.7234 | 0.0870 | 4.7193 | -2.9849 |
| 42 | Δ MADRS ~ EFctx.lh.precentral | 1.3961 | 0.1649 | 5.5618 | -3.0584 |
| 43 | Δ MADRS ~ EFctx.lh.precuneus | 1.7322 | 0.0854 | 5.1261 | -3.0170 |
| 44 | Δ MADRS ~ EFctx.lh.rostralanteriorcingulate | 0.6893 | 0.4918 | 5.6371 | -2.9984 |
| 45 | Δ MADRS ~ EFctx.lh.rostralmiddlefrontal | 1.0314 | 0.3041 | 5.7001 | -2.9903 |
| 46 | Δ MADRS ~ EFctx.lh.superiorfrontal | 1.7343 | 0.0851 | 5.4624 | -3.0974 |
| 47 | Δ MADRS ~ EFctx.lh.superiorparietal | 1.8563 | 0.0655 | 5.3965 | -3.1262 |
| 48 | Δ MADRS ~ EFctx.lh.superiortemporal | 1.2854 | 0.2008 | 5.7506 | -3.0322 |
| 49 | Δ MADRS ~ EFctx.lh.supramarginal | 0.9368 | 0.3505 | 5.6569 | -2.9983 |
| 50 | Δ MADRS ~ EFctx.lh.frontalpole | 1.5205 | 0.1306 | 5.8005 | -2.9350 |
| 51 | Δ MADRS ~ EFctx.lh.temporalpole | 0.0839 | 0.9333 | 5.5167 | -2.9689 |
| 52 | Δ MADRS ~ EFctx.lh.transversetemporal | 1.0350 | 0.3024 | 5.6356 | -2.9753 |
| 53 | Δ MADRS ~ EFctx.rh.bankssts | 0.2089 | 0.8348 | 5.5736 | -2.9477 |
| 54 | Δ MADRS ~ EFctx.rh.caudalanteriorcingulate | 1.3109 | 0.1920 | 5.5031 | -3.0585 |
| 55 | Δ MADRS ~ EFctx.rh.caudalmiddlefrontal | 0.9931 | 0.3224 | 5.3116 | -3.0101 |
| 56 | Δ MADRS ~ EFctx.rh.cuneus | 1.0257 | 0.3068 | 5.5313 | -2.9757 |
| 57 | Δ MADRS ~ EFctx.rh.entorhinal | 0.3188 | 0.7503 | 5.1370 | -2.9683 |
| 58 | Δ MADRS ~ EFctx.rh.fusiform | 0.0353 | 0.9719 | 5.3492 | -2.9627 |
| 59 | Δ MADRS ~ EFctx.rh.inferiorparietal | 0.4416 | 0.6594 | 5.5409 | -2.9719 |
| 60 | Δ MADRS ~ EFctx.rh.inferiortemporal | 0.0980 | 0.9220 | 5.5216 | -2.9687 |
| 61 | Δ MADRS ~ EFctx.rh.isthmuscingulate | 1.1468 | 0.2534 | 5.5553 | -3.0096 |
| 62 | Δ MADRS ~ EFctx.rh.lateraloccipital | 1.3549 | 0.1776 | 5.5079 | -2.9649 |
| 63 | Δ MADRS ~ EFctx.rh.lateralorbitofrontal | 0.1181 | 0.9061 | 5.4682 | -2.9482 |
| 64 | Δ MADRS ~ EFctx.rh.lingual | 0.4466 | 0.6558 | 5.4910 | -2.9775 |
| 65 | Δ MADRS ~ EFctx.rh.medialorbitofrontal | 0.8851 | 0.3776 | 5.1102 | -2.9038 |
| 66 | Δ MADRS ~ EFctx.rh.middletemporal | -0.1713 | 0.8642 | 5.5875 | -2.9728 |
| 67 | Δ MADRS ~ EFctx.rh.parahippocampal | -0.0533 | 0.9576 | 5.4170 | -2.9421 |
| 68 | Δ MADRS ~ EFctx.rh.paracentral | 1.4722 | 0.1432 | 5.2359 | -3.0446 |
| 69 | Δ MADRS ~ EFctx.rh.parsopercularis | 0.4057 | 0.6856 | 5.5058 | -2.9625 |
| 70 | Δ MADRS ~ EFctx.rh.parsorbitalis | -1.5217 | 0.1303 | 4.4952 | -3.2479 |
| 71 | Δ MADRS ~ EFctx.rh.parstriangularis | 0.0523 | 0.9584 | 5.5856 | -2.9713 |
| 72 | Δ MADRS ~ EFctx.rh.pericalcarine | 0.8017 | 0.4241 | 5.5964 | -2.9479 |
| 73 | Δ MADRS ~ EFctx.rh.postcentral | 0.8949 | 0.3723 | 5.2388 | -3.0063 |
| 74 | Δ MADRS ~ EFctx.rh.posteriorcingulate | 1.8661 | 0.0641 | 4.5850 | -2.9572 |
| 75 | Δ MADRS ~ EFctx.rh.precentral | 0.4125 | 0.6806 | 5.3439 | -2.9885 |
| 76 | Δ MADRS ~ EFctx.rh.precuneus | 1.5202 | 0.1307 | 5.1634 | -2.9922 |
| 77 | Δ MADRS ~ EFctx.rh.rostralanteriorcingulate | 0.7513 | 0.4537 | 5.6464 | -2.9519 |
| 78 | Δ MADRS ~ EFctx.rh.rostralmiddlefrontal | 0.2203 | 0.8259 | 5.5770 | -2.9690 |
| 79 | Δ MADRS ~ EFctx.rh.superiorfrontal | 1.5058 | 0.1344 | 5.3401 | -3.1130 |
| 80 | Δ MADRS ~ EFctx.rh.superiorparietal | 1.1193 | 0.2649 | 5.3231 | -3.0022 |
| 81 | Δ MADRS ~ EFctx.rh.superiortemporal | 0.5845 | 0.5598 | 5.4082 | -2.9776 |
| 82 | Δ MADRS ~ EFctx.rh.supramarginal | 0.5194 | 0.6043 | 5.4970 | -2.9756 |
| 83 | Δ MADRS ~ EFctx.rh.frontalpole | 1.6027 | 0.1112 | 5.5188 | -2.9469 |
| 84 | Δ MADRS ~ EFctx.rh.temporalpole | -0.4055 | 0.6857 | 5.6013 | -2.9909 |
| 85 | Δ MADRS ~ EFctx.rh.transversetemporal | 0.7262 | 0.4689 | 5.5628 | -2.9155 |

The relationship between clinical response (absolute change) and EF across individuals (Δ MADRS ~ EF + Baseline MADRS + Age + ECTnum + Site)

|  | roi | tEF | pEF | t_BasMADRS_ | tAge | tECTnum |
| --- | --- | --- | --- | --- | --- | --- |
| 1 | Δ MADRS ~ EFLeft.Cerebellum.Cortex | 1.870 | 0.064 | 9.723 | 6.138 | -3.104 |
| 2 | Δ MADRS ~ EFLeft.Thalamus.Proper | 1.520 | 0.131 | 9.712 | 6.441 | -3.187 |
| 3 | Δ MADRS ~ EFLeft.Caudate | 0.895 | 0.372 | 9.573 | 6.029 | -3.121 |
| 4 | Δ MADRS ~ EFLeft.Putamen | 1.485 | 0.140 | 9.684 | 6.588 | -3.154 |
| 5 | Δ MADRS ~ EFLeft.Pallidum | 1.779 | 0.077 | 9.769 | 6.664 | -3.173 |
| 6 | Δ MADRS ~ EFBrain.Stem | 1.620 | 0.108 | 9.707 | 6.347 | -3.060 |
| 7 | Δ MADRS ~ EFLeft.Hippocampus | 1.730 | 0.086 | 9.719 | 6.560 | -3.054 |
| 8 | Δ MADRS ~ EFLeft.Amygdala | 1.373 | 0.172 | 9.604 | 6.502 | -3.074 |
| 9 | Δ MADRS ~ EFLeft.Accumbens.area | 1.483 | 0.140 | 9.677 | 6.328 | -3.203 |
| 10 | Δ MADRS ~ EFLeft.VentralDC | 1.672 | 0.097 | 9.755 | 6.555 | -3.085 |
| 11 | Δ MADRS ~ EFRight.Cerebellum.Cortex | 1.592 | 0.114 | 9.712 | 6.218 | -3.102 |
| 12 | Δ MADRS ~ EFRight.Thalamus.Proper | 1.208 | 0.229 | 9.642 | 6.048 | -3.157 |
| 13 | Δ MADRS ~ EFRight.Caudate | 0.810 | 0.419 | 9.557 | 5.744 | -3.101 |
| 14 | Δ MADRS ~ EFRight.Putamen | 0.959 | 0.339 | 9.567 | 5.870 | -3.081 |
| 15 | Δ MADRS ~ EFRight.Pallidum | 1.034 | 0.303 | 9.586 | 5.893 | -3.122 |
| 16 | Δ MADRS ~ EFRight.Hippocampus | 1.153 | 0.251 | 9.596 | 5.639 | -3.043 |
| 17 | Δ MADRS ~ EFRight.Amygdala | 0.656 | 0.513 | 9.517 | 5.560 | -3.046 |
| 18 | Δ MADRS ~ EFRight.Accumbens.area | 0.594 | 0.554 | 9.516 | 5.338 | -3.108 |
| 19 | Δ MADRS ~ EFRight.VentralDC | 1.338 | 0.183 | 9.676 | 6.182 | -3.077 |
| 20 | Δ MADRS ~ EFctx.lh.bankssts | 1.428 | 0.155 | 9.663 | 6.605 | -3.052 |
| 21 | Δ MADRS ~ EFctx.lh.caudalanteriorcingulate | 1.262 | 0.209 | 9.584 | 5.569 | -3.176 |
| 22 | Δ MADRS ~ EFctx.lh.caudalmiddlefrontal | 2.177 | 0.031 | 9.765 | 5.867 | -3.237 |
| 23 | Δ MADRS ~ EFctx.lh.cuneus | 1.466 | 0.145 | 9.558 | 6.416 | -3.105 |
| 24 | Δ MADRS ~ EFctx.lh.entorhinal | 1.262 | 0.209 | 9.618 | 5.542 | -3.187 |
| 25 | Δ MADRS ~ EFctx.lh.fusiform | 1.303 | 0.195 | 9.669 | 6.098 | -3.119 |
| 26 | Δ MADRS ~ EFctx.lh.inferiorparietal | 1.646 | 0.102 | 9.600 | 6.463 | -3.150 |
| 27 | Δ MADRS ~ EFctx.lh.inferiortemporal | 1.285 | 0.201 | 9.641 | 6.514 | -3.090 |
| 28 | Δ MADRS ~ EFctx.lh.isthmuscingulate | 1.810 | 0.072 | 9.716 | 6.411 | -3.126 |
| 29 | Δ MADRS ~ EFctx.lh.lateraloccipital | 1.272 | 0.206 | 9.603 | 6.390 | -3.113 |
| 30 | Δ MADRS ~ EFctx.lh.lateralorbitofrontal | 1.484 | 0.140 | 9.543 | 6.096 | -3.085 |
| 31 | Δ MADRS ~ EFctx.lh.lingual | 0.297 | 0.767 | 9.509 | 5.662 | -3.090 |
| 32 | Δ MADRS ~ EFctx.lh.medialorbitofrontal | 1.221 | 0.224 | 9.507 | 5.689 | -3.044 |
| 33 | Δ MADRS ~ EFctx.lh.middletemporal | 1.943 | 0.054 | 9.705 | 6.651 | -3.153 |
| 34 | Δ MADRS ~ EFctx.lh.parahippocampal | 1.764 | 0.080 | 9.772 | 6.042 | -3.104 |
| 35 | Δ MADRS ~ EFctx.lh.paracentral | 2.112 | 0.036 | 9.807 | 5.461 | -3.253 |
| 36 | Δ MADRS ~ EFctx.lh.parsopercularis | 1.234 | 0.219 | 9.599 | 6.035 | -3.118 |
| 37 | Δ MADRS ~ EFctx.lh.parsorbitalis | 0.123 | 0.903 | 9.467 | 5.486 | -3.100 |
| 38 | Δ MADRS ~ EFctx.lh.parstriangularis | 0.967 | 0.335 | 9.539 | 5.872 | -3.121 |
| 39 | Δ MADRS ~ EFctx.lh.pericalcarine | 0.269 | 0.788 | 9.498 | 5.761 | -3.075 |
| 40 | Δ MADRS ~ EFctx.lh.postcentral | 1.883 | 0.062 | 9.642 | 5.828 | -3.161 |
| 41 | Δ MADRS ~ EFctx.lh.posteriorcingulate | 2.204 | 0.029 | 9.824 | 5.189 | -3.121 |
| 42 | Δ MADRS ~ EFctx.lh.precentral | 1.867 | 0.064 | 9.715 | 5.827 | -3.199 |
| 43 | Δ MADRS ~ EFctx.lh.precuneus | 2.233 | 0.027 | 9.766 | 5.956 | -3.180 |
| 44 | Δ MADRS ~ EFctx.lh.rostralanteriorcingulate | 0.839 | 0.403 | 9.478 | 6.020 | -3.114 |
| 45 | Δ MADRS ~ EFctx.lh.rostralmiddlefrontal | 1.423 | 0.157 | 9.585 | 6.170 | -3.109 |
| 46 | Δ MADRS ~ EFctx.lh.superiorfrontal | 2.271 | 0.025 | 9.775 | 5.677 | -3.269 |
| 47 | Δ MADRS ~ EFctx.lh.superiorparietal | 2.103 | 0.037 | 9.708 | 5.808 | -3.253 |
| 48 | Δ MADRS ~ EFctx.lh.superiortemporal | 1.783 | 0.077 | 9.714 | 6.681 | -3.158 |
| 49 | Δ MADRS ~ EFctx.lh.supramarginal | 1.395 | 0.165 | 9.636 | 6.367 | -3.115 |
| 50 | Δ MADRS ~ EFctx.lh.frontalpole | 1.643 | 0.103 | 9.470 | 5.968 | -3.045 |
| 51 | Δ MADRS ~ EFctx.lh.temporalpole | 0.229 | 0.819 | 9.499 | 5.629 | -3.114 |
| 52 | Δ MADRS ~ EFctx.lh.transversetemporal | 1.187 | 0.237 | 9.605 | 6.133 | -3.083 |
| 53 | Δ MADRS ~ EFctx.rh.bankssts | 0.688 | 0.492 | 9.559 | 6.134 | -3.019 |
| 54 | Δ MADRS ~ EFctx.rh.caudalanteriorcingulate | 1.400 | 0.164 | 9.552 | 5.990 | -3.171 |
| 55 | Δ MADRS ~ EFctx.rh.caudalmiddlefrontal | 1.109 | 0.269 | 9.591 | 5.573 | -3.136 |
| 56 | Δ MADRS ~ EFctx.rh.cuneus | 1.375 | 0.171 | 9.581 | 6.249 | -3.077 |
| 57 | Δ MADRS ~ EFctx.rh.entorhinal | 0.507 | 0.613 | 9.504 | 5.241 | -3.098 |
| 58 | Δ MADRS ~ EFctx.rh.fusiform | 0.538 | 0.591 | 9.533 | 5.698 | -3.044 |
| 59 | Δ MADRS ~ EFctx.rh.inferiorparietal | 1.132 | 0.260 | 9.623 | 6.095 | -3.083 |
| 60 | Δ MADRS ~ EFctx.rh.inferiortemporal | 0.134 | 0.894 | 9.487 | 5.637 | -3.101 |
| 61 | Δ MADRS ~ EFctx.rh.isthmuscingulate | 1.704 | 0.091 | 9.737 | 6.355 | -3.132 |
| 62 | Δ MADRS ~ EFctx.rh.lateraloccipital | 1.877 | 0.063 | 9.694 | 6.344 | -3.065 |
| 63 | Δ MADRS ~ EFctx.rh.lateralorbitofrontal | 0.633 | 0.528 | 9.536 | 5.665 | -3.024 |
| 64 | Δ MADRS ~ EFctx.rh.lingual | 1.323 | 0.188 | 9.670 | 6.268 | -3.094 |
| 65 | Δ MADRS ~ EFctx.rh.medialorbitofrontal | 1.287 | 0.200 | 9.635 | 5.588 | -2.983 |
| 66 | Δ MADRS ~ EFctx.rh.middletemporal | -0.322 | 0.748 | 9.477 | 5.551 | -3.115 |
| 67 | Δ MADRS ~ EFctx.rh.parahippocampal | 0.801 | 0.424 | 9.491 | 5.745 | -3.029 |
| 68 | Δ MADRS ~ EFctx.rh.paracentral | 1.915 | 0.058 | 9.760 | 5.274 | -3.184 |
| 69 | Δ MADRS ~ EFctx.rh.parsopercularis | 0.659 | 0.511 | 9.539 | 5.763 | -3.077 |
| 70 | Δ MADRS ~ EFctx.rh.parsorbitalis | -1.594 | 0.113 | 9.648 | 5.090 | -3.369 |
| 71 | Δ MADRS ~ EFctx.rh.parstriangularis | 0.018 | 0.986 | 9.494 | 5.691 | -3.105 |
| 72 | Δ MADRS ~ EFctx.rh.pericalcarine | 1.313 | 0.191 | 9.576 | 6.437 | -3.033 |
| 73 | Δ MADRS ~ EFctx.rh.postcentral | 0.938 | 0.350 | 9.541 | 5.473 | -3.128 |
| 74 | Δ MADRS ~ EFctx.rh.posteriorcingulate | 2.369 | 0.019 | 9.857 | 5.169 | -3.101 |
| 75 | Δ MADRS ~ EFctx.rh.precentral | 0.608 | 0.544 | 9.511 | 5.472 | -3.128 |
| 76 | Δ MADRS ~ EFctx.rh.precuneus | 2.185 | 0.031 | 9.818 | 5.745 | -3.138 |
| 77 | Δ MADRS ~ EFctx.rh.rostralanteriorcingulate | 1.015 | 0.312 | 9.508 | 6.214 | -3.047 |
| 78 | Δ MADRS ~ EFctx.rh.rostralmiddlefrontal | 0.603 | 0.548 | 9.539 | 5.937 | -3.082 |
| 79 | Δ MADRS ~ EFctx.rh.superiorfrontal | 1.938 | 0.055 | 9.739 | 5.323 | -3.282 |
| 80 | Δ MADRS ~ EFctx.rh.superiorparietal | 1.513 | 0.133 | 9.656 | 5.638 | -3.128 |
| 81 | Δ MADRS ~ EFctx.rh.superiortemporal | 0.621 | 0.536 | 9.526 | 5.792 | -3.093 |
| 82 | Δ MADRS ~ EFctx.rh.supramarginal | 0.941 | 0.348 | 9.587 | 5.955 | -3.090 |
| 83 | Δ MADRS ~ EFctx.rh.frontalpole | 1.613 | 0.109 | 9.364 | 5.225 | -3.073 |
| 84 | Δ MADRS ~ EFctx.rh.temporalpole | -0.285 | 0.776 | 9.490 | 5.647 | -3.119 |
| 85 | Δ MADRS ~ EFctx.rh.transversetemporal | 1.129 | 0.261 | 9.615 | 6.142 | -2.995 |
