# Supplementary material for: Electric field causes volumetric changes in the human brain
Source: eLife. 2019 Oct 23;8:e49115. doi: 10.7554/eLife.49115 (PMC6874416; doi:10.7554/eLife.49115)
Supplement: Supplementary file 5. [file elife-49115-supp5.docx]

The relationship between baseline volume and age (Baseline Volume ~ Age)

|  | roi | tAge | pAge |
| --- | --- | --- | --- |
| 1 | Baseline VolLeft.Cerebellum.Cortex ~ Age | -4.2768 | 0.0000 |
| 2 | Baseline VolLeft.Thalamus.Proper ~ Age | -2.6668 | 0.0085 |
| 3 | Baseline VolLeft.Caudate ~ Age | 1.1587 | 0.2485 |
| 4 | Baseline VolLeft.Putamen ~ Age | -5.1151 | 0.0000 |
| 5 | Baseline VolLeft.Pallidum ~ Age | -2.0723 | 0.0400 |
| 6 | Baseline VolBrain.Stem ~ Age | -1.7714 | 0.0786 |
| 7 | Baseline VolLeft.Hippocampus ~ Age | -6.5427 | 0.0000 |
| 8 | Baseline VolLeft.Amygdala ~ Age | -4.6348 | 0.0000 |
| 9 | Baseline VolLeft.Accumbens.area ~ Age | -4.6046 | 0.0000 |
| 10 | Baseline VolLeft.VentralDC ~ Age | -1.3501 | 0.1791 |
| 11 | Baseline VolRight.Cerebellum.Cortex ~ Age | -4.1066 | 0.0001 |
| 12 | Baseline VolRight.Thalamus.Proper ~ Age | -3.6386 | 0.0004 |
| 13 | Baseline VolRight.Caudate ~ Age | -1.2939 | 0.1978 |
| 14 | Baseline VolRight.Putamen ~ Age | -6.7879 | 0.0000 |
| 15 | Baseline VolRight.Pallidum ~ Age | -4.5543 | 0.0000 |
| 16 | Baseline VolRight.Hippocampus ~ Age | -5.6416 | 0.0000 |
| 17 | Baseline VolRight.Amygdala ~ Age | -3.6549 | 0.0004 |
| 18 | Baseline VolRight.Accumbens.area ~ Age | -5.8783 | 0.0000 |
| 19 | Baseline VolRight.VentralDC ~ Age | -2.5733 | 0.0111 |
| 20 | Baseline Volctx.lh.bankssts ~ Age | -2.8936 | 0.0044 |
| 21 | Baseline Volctx.lh.caudalanteriorcingulate ~ Age | -0.8132 | 0.4174 |
| 22 | Baseline Volctx.lh.caudalmiddlefrontal ~ Age | -3.3319 | 0.0011 |
| 23 | Baseline Volctx.lh.cuneus ~ Age | -2.9607 | 0.0036 |
| 24 | Baseline Volctx.lh.entorhinal ~ Age | -3.5206 | 0.0006 |
| 25 | Baseline Volctx.lh.fusiform ~ Age | -5.5547 | 0.0000 |
| 26 | Baseline Volctx.lh.inferiorparietal ~ Age | -4.1748 | 0.0001 |
| 27 | Baseline Volctx.lh.inferiortemporal ~ Age | -5.6376 | 0.0000 |
| 28 | Baseline Volctx.lh.isthmuscingulate ~ Age | -3.3345 | 0.0011 |
| 29 | Baseline Volctx.lh.lateraloccipital ~ Age | -3.7491 | 0.0003 |
| 30 | Baseline Volctx.lh.lateralorbitofrontal ~ Age | -7.0033 | 0.0000 |
| 31 | Baseline Volctx.lh.lingual ~ Age | -5.9806 | 0.0000 |
| 32 | Baseline Volctx.lh.medialorbitofrontal ~ Age | -1.9891 | 0.0486 |
| 33 | Baseline Volctx.lh.middletemporal ~ Age | -6.2512 | 0.0000 |
| 34 | Baseline Volctx.lh.parahippocampal ~ Age | -7.4016 | 0.0000 |
| 35 | Baseline Volctx.lh.paracentral ~ Age | -1.9326 | 0.0553 |
| 36 | Baseline Volctx.lh.parsopercularis ~ Age | -5.0408 | 0.0000 |
| 37 | Baseline Volctx.lh.parsorbitalis ~ Age | -5.3290 | 0.0000 |
| 38 | Baseline Volctx.lh.parstriangularis ~ Age | -5.5008 | 0.0000 |
| 39 | Baseline Volctx.lh.pericalcarine ~ Age | -2.0474 | 0.0424 |
| 40 | Baseline Volctx.lh.postcentral ~ Age | -5.1513 | 0.0000 |
| 41 | Baseline Volctx.lh.posteriorcingulate ~ Age | -3.4563 | 0.0007 |
| 42 | Baseline Volctx.lh.precentral ~ Age | -4.3857 | 0.0000 |
| 43 | Baseline Volctx.lh.precuneus ~ Age | -4.0321 | 0.0001 |
| 44 | Baseline Volctx.lh.rostralanteriorcingulate ~ Age | -2.3314 | 0.0211 |
| 45 | Baseline Volctx.lh.rostralmiddlefrontal ~ Age | -3.9878 | 0.0001 |
| 46 | Baseline Volctx.lh.superiorfrontal ~ Age | -5.4678 | 0.0000 |
| 47 | Baseline Volctx.lh.superiorparietal ~ Age | -4.8697 | 0.0000 |
| 48 | Baseline Volctx.lh.superiortemporal ~ Age | -6.6056 | 0.0000 |
| 49 | Baseline Volctx.lh.supramarginal ~ Age | -5.6008 | 0.0000 |
| 50 | Baseline Volctx.lh.frontalpole ~ Age | -3.7056 | 0.0003 |
| 51 | Baseline Volctx.lh.temporalpole ~ Age | -3.3124 | 0.0012 |
| 52 | Baseline Volctx.lh.transversetemporal ~ Age | -5.2842 | 0.0000 |
| 53 | Baseline Volctx.rh.bankssts ~ Age | -5.8200 | 0.0000 |
| 54 | Baseline Volctx.rh.caudalanteriorcingulate ~ Age | -0.1144 | 0.9091 |
| 55 | Baseline Volctx.rh.caudalmiddlefrontal ~ Age | -3.7977 | 0.0002 |
| 56 | Baseline Volctx.rh.cuneus ~ Age | -2.8824 | 0.0046 |
| 57 | Baseline Volctx.rh.entorhinal ~ Age | -1.8970 | 0.0598 |
| 58 | Baseline Volctx.rh.fusiform ~ Age | -5.2218 | 0.0000 |
| 59 | Baseline Volctx.rh.inferiorparietal ~ Age | -5.7134 | 0.0000 |
| 60 | Baseline Volctx.rh.inferiortemporal ~ Age | -4.6609 | 0.0000 |
| 61 | Baseline Volctx.rh.isthmuscingulate ~ Age | -4.2929 | 0.0000 |
| 62 | Baseline Volctx.rh.lateraloccipital ~ Age | -3.7913 | 0.0002 |
| 63 | Baseline Volctx.rh.lateralorbitofrontal ~ Age | -4.8756 | 0.0000 |
| 64 | Baseline Volctx.rh.lingual ~ Age | -5.3444 | 0.0000 |
| 65 | Baseline Volctx.rh.medialorbitofrontal ~ Age | -3.0897 | 0.0024 |
| 66 | Baseline Volctx.rh.middletemporal ~ Age | -6.9557 | 0.0000 |
| 67 | Baseline Volctx.rh.parahippocampal ~ Age | -5.6025 | 0.0000 |
| 68 | Baseline Volctx.rh.paracentral ~ Age | -2.0546 | 0.0417 |
| 69 | Baseline Volctx.rh.parsopercularis ~ Age | -5.4711 | 0.0000 |
| 70 | Baseline Volctx.rh.parsorbitalis ~ Age | -5.0994 | 0.0000 |
| 71 | Baseline Volctx.rh.parstriangularis ~ Age | -4.4944 | 0.0000 |
| 72 | Baseline Volctx.rh.pericalcarine ~ Age | -1.7874 | 0.0760 |
| 73 | Baseline Volctx.rh.postcentral ~ Age | -3.7190 | 0.0003 |
| 74 | Baseline Volctx.rh.posteriorcingulate ~ Age | -3.6034 | 0.0004 |
| 75 | Baseline Volctx.rh.precentral ~ Age | -3.9917 | 0.0001 |
| 76 | Baseline Volctx.rh.precuneus ~ Age | -5.6244 | 0.0000 |
| 77 | Baseline Volctx.rh.rostralanteriorcingulate ~ Age | -1.7497 | 0.0823 |
| 78 | Baseline Volctx.rh.rostralmiddlefrontal ~ Age | -4.5143 | 0.0000 |
| 79 | Baseline Volctx.rh.superiorfrontal ~ Age | -6.1004 | 0.0000 |
| 80 | Baseline Volctx.rh.superiorparietal ~ Age | -3.9167 | 0.0001 |
| 81 | Baseline Volctx.rh.superiortemporal ~ Age | -8.2332 | 0.0000 |
| 82 | Baseline Volctx.rh.supramarginal ~ Age | -5.8496 | 0.0000 |
| 83 | Baseline Volctx.rh.frontalpole ~ Age | -2.3721 | 0.0190 |
| 84 | Baseline Volctx.rh.temporalpole ~ Age | -1.6448 | 0.1022 |
| 85 | Baseline Volctx.rh.transversetemporal ~ Age | -3.8244 | 0.0002 |
