# Supplementary material for: Electric field causes volumetric changes in the human brain
Source: eLife. 2019 Oct 23;8:e49115. doi: 10.7554/eLife.49115 (PMC6874416; doi:10.7554/eLife.49115)
Supplement: Supplementary file 6. — The table indicates the parameters of the structural image acquisition across sites. [file elife-49115-supp6.docx]

MRI Summary

| Site | TESLA | TR (ms) | TE (ms) | Flip Angle | Voxel-size (mm) |
| --- | --- | --- | --- | --- | --- |
| 1 | 3 | 2530 | 1.74-7.32 | 7 | 1.3x1.0x1.0 |
| 2 | 3 | 2530 | 1.64-9.08 | 7 | 1.0x1.0x1.0 |
| 3 | 3 | 7.84 | 3.02 | 12 | 0.94x0.94x1.0 |
| 4 | 3 | 7.4 | 3.4 | 9 | 0.5x0.5x0.5 |
| 5 | 3 | 7.83 | 3.02 | 8 | 0.94x0.94x1.0 |
| 6 | 3 | 6.7 | 2.9 | 8 | 1.0x1.0x1.0 |
| 7 | 3 | 9600 | 4.6 | 8 | 0.98x0.98x1.2 |
