# Supplementary material for: Electric field causes volumetric changes in the human brain
Source: eLife. 2019 Oct 23;8:e49115. doi: 10.7554/eLife.49115 (PMC6874416; doi:10.7554/eLife.49115)
Supplement: Transparent reporting form [file elife-49115-transrepform.pdf]

eLIFE
1st Floor
24 Hills Road
Cambridge CB2 1JP, UK
P 01223 855340
W elifesciences.org
T @elife

eLife's transparent reporting form

We encourage authors to provide detailed information *within their submission* to facilitate the interpretation and replication of experiments. Authors can upload supporting documentation to indicate the use of appropriate reporting guidelines for health-related research (see EQUATOR Network), life science research (see the BioSharing Information Resource), or the ARRIVE guidelines for reporting work involving animal research. Where applicable, authors should refer to any relevant reporting standards documents in this form.

If you have any questions, please consult our Journal Policies and/or contact us: editorial@elifesciences.org.

Sample-size estimation

- You should state whether an appropriate sample size was computed when the study was being designed
- You should state the statistical method of sample size computation and any required assumptions
- If no explicit power analysis was used, you should describe how you decided what sample (replicate) size (number) to use

Please outline where this information can be found within the submission (e.g., sections or figure legends), or explain why this information doesn't apply to your submission:

In an earlier manuscript (Oltedal et al. 2017 Neuroimage: Clinical) from the GEMRIC consortium, we published our effect size estimations for volume changes based on the samples from one site. Medial temporal lobe structure volume changes had Cohen's d over 1.0 and it meant that N~20 is necessary to have 80% power with $\alpha=0.01$. The volume changes of other cortical and subcortical areas with Cohen's d>0.5 needed N>31. These results indicated robust volume changes during ECT and our study with its large sample size (N=151) is well powered to detect volume changes. The relationship between volume change and electric field is novel, and no apriori estimation was possible.

Replicates

- You should report how often each experiment was performed
- You should include a definition of biological versus technical replication
- The data obtained should be provided and sufficient information should be provided to indicate the number of independent biological and/or technical replicates
- If you encountered any outliers, you should describe how these were handled
- Criteria for exclusion/inclusion of data should be clearly stated
- High-throughput sequence data should be uploaded before submission, with a private link for reviewers provided (these are available from both GEO and ArrayExpress)

Please outline where this information can be found within the submission (e.g., sections or figure legends), or explain why this information doesn't apply to your submission:

eLife Sciences Publications, Ltd is a limited liability non-profit non-stock corporation incorporated in the State of Delaware, USA, with company number 5030732, and is registered in the UK with company number FC030576 and branch number BR015634 at the address 1st Floor, 24 Hills Road, Cambridge CB2 1JP | August 2014

This is a longitudinal neuroimaging study, each individual was scanned twice, before and after ECT.

Statistical reporting

- Statistical analysis methods should be described and justified
- Raw data should be presented in figures whenever informative to do so (typically when N per group is less than 10)
- For each experiment, you should identify the statistical tests used, exact values of N, definitions of center, methods of multiple test correction, and dispersion and precision measures (e.g., mean, median, SD, SEM, confidence intervals; and, for the major substantive results, a measure of effect size (e.g., Pearson's r, Cohen's d)
- Report exact p-values wherever possible alongside the summary statistics and 95% confidence intervals. These should be reported for all key questions and not only when the p-value is less than 0.05.

Please outline where this information can be found within the submission (e.g., sections or figure legends), or explain why this information doesn't apply to your submission:

The data was collected at 7 different sites, two times before and after electroconvulsive therapy. Our mixed effect model treated sites as random variable. We had N=151 individuals before and after ECT.
First we compared average EF and average volume changes across 85 ROIs and found strong correlation ($t = 3.77$, $df = 83$, $r = 0.38$, $p = 0.0003$).
Second, we compared individual EF and volume changes in 85 distinct regions. We performed three regressions:
1) $\Delta Vol \sim EF + Age + \text{number of ECT sessions} + site$
2) $\Delta MADRS \sim \Delta Vol + Age + \text{number of ECT sessions} + site$
3) $\Delta MADRS \sim EF + Age + \text{number of ECT sessions} + site$;
where EF, age, and the number of ECT sessions were fixed effects, and the site was a random effect, while the dependent variable was volume change and clinical response (MADRS scale change) respectively. Age, number of ECT sessions, and site, considered as nuisance variables, were included based on our prior observations of an inverse relationship between ECT session number and response. Further, age is also shown to impact clinical response (older patients have increased the probability of response, in our sample: $t = 5.75$, $df = 149$, $r = 0.43$, $p < 10^{-7}$) and age-related changes on brain structure are related to EF. We used Benjamini and Hochberg false discovery rate (FDR) correction method to control for multiple comparisons across 85 ROIs, where a conservative FDR corrected $p < 0.01$ was chosen as the statistical threshold of significance.
In analysis 1) we found two areas: left amygdala and hippocampus to survive FDR correction, hippocampus: $t = 4.5$, FDR corrected $p = 0.001$, amygdala: $t = 3.9$, FDR corrected $p = 0.006$. In analysis 2) and 3) we found no areas with significant results.

(For large datasets, or papers with a very large number of statistical tests, you may upload a single table file with tests, Ns, etc., with reference to sections in the manuscript.)

Group allocation

- Indicate how samples were allocated into experimental groups (in the case of clinical studies, please specify allocation to treatment method); if randomization was used, please also state if restricted randomization was applied
- Indicate if masking was used during group allocation, data collection and/or data analysis

Please outline where this information can be found within the submission (e.g., sections or figure legends), or explain why this information doesn't apply to your submission:

This is a longitudinal neuroimaging study with one cohort of patients with major depressive episode, who had an underlying diagnosis of either bipolar (N=12) and major depression (N=139).

Additional data files ("source data")

- We encourage you to upload relevant additional data files, such as numerical data that are represented as a graph in a figure, or as a summary table
- Where provided, these should be in the most useful format, and they can be uploaded as "Source data" files linked to a main figure or table
- Include model definition files including the full list of parameters used
- Include code used for data analysis (e.g., R, MatLab)
- Avoid stating that data files are "available upon request"

Please indicate the figures or tables for which source data files have been provided:

We upload our analysis file (R scripts in org mode) and also made it available on github (link is in the manuscript). Also the EF modeling docker image is available at hub.docker.com amiklos/roast:1.1105
